# Maternal characteristics and outcomes affected by hypothyroidism during pregnancy (maternal hypothyroidism on pregnancy outcomes, MHPO-1)

**DOI:** 10.1186/s12884-019-2596-9

**Published:** 2019-12-05

**Authors:** Zareen Kiran, Aisha Sheikh, Sarwar Malik, Areeba Meraj, Maha Masood, Safana Ismail, Muhammad Owais Rashid, Quratulain Shaikh, Numan Majeed, Luman Sheikh, Najmul Islam

**Affiliations:** 1 Section of Endocrinology, Department of Medicine, Aga Khan University Hospital, Stadium Road, P.O. Box 3500, Karachi, Pakistan; 2 Department of Endocrinology, Ali Medical Center, Islamabad, Pakistan; 3 Dow Medical College, Dow University of Health Sciences, Karachi, Pakistan; 4 Karachi Medical & Dental College, Karachi, Pakistan; 5 Indus Hospital Research Center, Karachi, Pakistan; 6 Department of Chemical Pathology, Army Medical College, Rawalpindi, Pakistan; 7 Department of Obstetrics & Gynecology, Aga Khan University Hospital, Karachi, Pakistan

**Keywords:** Thyroid disorders, Preconception, Conception, Complications, Effects, Management

## Abstract

**Background:**

Hypothyroidism in pregnancy is an arena of ongoing research, with international conflicts regarding screening, management, and outcomes. Various studies have described the outcomes depending on geographical and international diagnostic criteria. No study has been conducted in this regard from the region of Pakistan. Therefore, we aim to report the clinical features and maternal outcomes of hypothyroid pregnancies and compare the maternal outcomes between uncontrolled and controlled TSH levels in the preconception as well as the gestational period.

**Methods:**

We conducted a cross-sectional retrospective study on 718 cases in the Aga Khan University Hospital after ethical approval. We collected information on pregnant females who have diagnosed hypothyroidism before conception or during their antenatal period. We noted the maternal characteristics and maternal comorbidities. Laboratory data were recorded for thyroid stimulating hormone levels before conception and during gestation. We recorded maternal outcomes as pregnancy loss (including miscarriage, stillbirth/intrauterine death, medical termination of pregnancy and ectopic pregnancy), gestational hypertension, pre-eclampsia, postpartum hemorrhage, placental abruption, and modalities of delivery. Data analysis was performed on Statistical Package for the Social Sciences version 20.0.

**Results:**

Among 708 hypothyroid women 638 had live births. Postpartum hemorrhage was the most frequent maternal outcome (38.8%). The emergency cesarean section occurred in 23.4% of cases. We determined TSH levels in 53.2, 56.7, 61.7 and 66.6% of cases in preconception, 1st, 2nd, and 3rd trimester periods. A significant association existed between cesarean section and preconception thyrotropin levels > 2.5 mIU/L, whereas postpartum hemorrhage was significantly associated with thyrotropin levels > 2.5 mIU/L in the preconception and third trimester.

**Conclusion:**

Successful live births in our patients were complicated by maternal postpartum hemorrhage and a frequent number of emergency cesarean section.

## Background

One of the commonest endocrine disorders in women of reproductive age is hypothyroidism, which often presents as an inter-current disease during pregnancy and in the puerperium. Similar to the non-pregnant state; overt hypothyroidism in pregnancy is also defined as increased serum thyroid stimulating hormone (TSH) and decreased serum free thyroxine (FT4), which ranges in prevalence from 0.3–3% of pregnancies in western world [1–3], whereas recent studies from some countries of the Asian subcontinent have reported a higher but variable prevalence of 4.8% to up to 13.13% [4–7].

On the other hand, subclinical hypothyroidism (without typical symptoms of hypothyroidism, increased TSH and normal thyroid hormone levels), has an estimated prevalence of 2–5% of all cases reported in the literature [3, 8, 9].

There are several obstetric complications associated with hypothyroidism in pregnancy like gestational hypertension and miscarriages [10–12]. As thyroid hormones have many effects on cardiovascular physiology and blood pressure regulation, there is a higher prevalence of gestational hypertension compared to euthyroid women [13–17]. Moreover, inadequate thyroid hormone levels in the mother have also been associated with low birth weight and fetal death or abortions [12, 18]. Nevertheless, data in the past have suggested impaired neurocognitive development in fetuses and children of hypothyroid and euthyroid antibody-positive mothers [2, 19, 20], but recent trials have shown no benefit of treatment on the outcomes [21–23]. Maternal thyroid hormones also have a significant physiological role in early placental development by regulating human trophoblast proliferation and invasion [3, 8, 9, 19, 20]​. Inadequate trophoblast cell invasion may result in abnormal placentation (which is a notable risk factor for preterm delivery) [24–28]​ and placental abruption which can also lead to stillbirth [14]. Moreover, euthyroid women with autoimmune thyroid disease show impaired thyroid function during gestation and seem to suffer from a high rate of obstetrical complications [29]. Finally, populations’ urinary iodine excretion cannot be ignored. In 2011, National Nutrition Health Survey was conducted in Pakistan, according to which there is 48% iodine deficiency which has however improved over the last decade [30].

Thus, hypothyroidism is a serious, yet manageable medical disorder in pregnancy, and despite significant evidence of hypothyroid related maternal and neonatal complications, universal screening is currently not recommended in pregnant women or women of reproductive age by various professional societies.

To the best of our knowledge, maternal outcomes of pregnancies affected by hypothyroidism have never been reported from Pakistan. Therefore, we aim to report the clinical features and maternal outcomes of hypothyroid pregnancies and compare the maternal outcomes between uncontrolled and controlled TSH levels in the preconception as well as the gestational period.

## Methods

### Study design

We conducted a retrospective chart review of hypothyroid pregnant patients from their preconception to complete gestational phase (with whatever outcome), from 2008 to 2016, presenting to Aga Khan University, Karachi, Pakistan.

### Study setting

The hypothyroid pregnant females presenting to endocrine and obstetric clinics at the Aga Khan University Hospital.

#### Eligibility criteria

##### Inclusion criteria

We selected patients, according to the following inclusion criteria:

- All pregnant women attending endocrine and obstetric clinic who had been diagnosed hypothyroidism defined as either overt (elevated TSH and low FT4) or subclinical (elevated TSH and normal FT4) hypothyroidism and those labeled only ‘hypothyroidism’ (uncategorized) by the clinician either before or during pregnancy.
- These patients were also on levothyroxine replacement with doses adjusted appropriately to aim controlled hypothyroidism with TSH ≤2. 5 uIU/mL throughout pregnancy [31].

##### Exclusion criteria

Pregnant women who were not diagnosed as either overt or subclinical hypothyroidism or those not labeled ‘hypothyroidism’ were excluded from the study.

### Sample size assumption

The maternal outcomes of pregnancies affected by hypothyroidism, from literature showed that spontaneous abortions were 15.48% [32] in patients with subclinical hypothyroidism. Therefore, taking the frequency of 16% with a 95% confidence level and a bound on error of ±6% the estimated sample size was 278 pregnant women.

### Sampling technique

Non-probability consecutive sampling. Purposive sampling was employed.

### Data collection

We reviewed the medical record files of pregnant females from their preconception phase to gestational period, during the years 2008–2016. We selected these females by applying coding words of hypothyroidism and pregnancy in the electronic medical record maintained according to the international classification of diseases (ICD) coding system in the Health Information and Management System (HIMS) department of our hospital. Data were collected by trained medical doctors. It was randomly double-checked and corroborated by the principal investigator. Data on maternal characteristics included age, weight, height, parity and week(s) of pregnancy at first visit. We calculated body mass index from the weight and height taken on first visit, any time during pregnancy. History of diabetes, hypertension and smoking status was also noted, and history of hypothyroidism was detailed as either subclinical, overt or uncategorized according to the inclusion criteria stated earlier. Underlying cause of hypothyroidism and family history of hypothyroidism was noted wherever stated. Gestational age at delivery, modes of labour and indications as well as the mode of delivery were noted, as per records of obstetric clinical pathway maintained in the files. Finally, we recorded maternal outcomes like pregnancy loss, gestational hypertension, pre-eclampsia, postpartum hemorrhage, placental abruption and mode of delivery for all pregnancies from obstetric discharge summaries.

### Thyroid stimulating hormone levels and thyroid antibodies

TSH levels available before conception and during each month throughout pregnancy were noted. TSH levels were assessed by Advia Centaur (Siemens Diagnostics), Chemiluminescence immunoassay. Specificity of the Advia Centaur third generation TSH assay was determined against hCG (human chorionic gonadotropin), FSH (follicle-stimulating hormone) & LH (luteinizing hormone) with no significant cross-reactivity observed. The functional sensitivity of the assay is 0.008 uIU/mL, with the reference range from 0.008–150 uIU/mL. Thyroid peroxidase (TPO) antibodies were assayed on Immulite 2000 (Siemens Diagnostics) using Chemiluminescence immunoassay technique.

### Definitions of maternal outcomes

We defined maternal outcomes as per internationally accepted criteria.

- Pregnancy loss: [1] *Spontaneous Abortion*; defined as loss of pregnancy at or before 20 weeks of gestation. [2] *Intrauterine death (IUD) or stillbirth*; defined as pregnancy loss occurring after 20 weeks of gestation [33, 34]. Other outcomes include ‘*Medical termination of pregnancy*’ (anytime during pregnancy) and ‘*Ectopic pregnancy*’ as determined by the clinician based on obstetric examination and investigations.
- Gestational hypertension (GH): Women started on anti-hypertensives during antenatal visits were labeled as gestational hypertension, a condition characterized by high blood pressure that develops after week 20 in pregnancy [35]. Others were labelled as ‘Chronic Hypertension’.
- Pre-eclampsia: Pregnant women characterized by both high blood pressure (> 140/90 mmHg) and proteinuria (≥ 0.3 g/24 h) that develops after 20 weeks of gestation in a previously normotensive woman [36].
- Postpartum Hemorrhage: Defined as a blood loss of 500 ml or more within 24 h after birth [37].
- Placental Abruption: Defined as the separation of the placenta from uterine lining any time after the 20th week of pregnancy.
- Modes of delivery: These were noted as emergency or elective cesarean sections, spontaneous vaginal delivery with or without episiotomy and finally instrumental delivery.

### Statistical analysis

#### Descriptive statistics

We performed descriptive analysis for demographic and clinical features of hypothyroid pregnant women as well as the maternal outcomes. Results are presented as mean (with standard deviation) for normally distributed data and as median (interquartile range) for skewed distribution of quantitative variables. For qualitative variables, we used frequency (percentage). We grouped BMI into underweight (< 18.5), normal (18.5–24.9), overweight (25–29.9) and obese (> = 30) categories. Age was categorized into different age ranges. Similarly, parity was categorized into nulliparous and multiparous groups. After excluding missing TSH values for each trimester and pre conceptional periods, we analyzed the total number of patients for baseline characteristics as well as maternal outcomes. (See Additional file 1 for further details.)

#### Inferential statistics

We categorized preconception TSH levels into a controlled group (TSH levels ≤2.5 uIU/mL) and uncontrolled group (TSH levels > 2.5 uIU/mL) as per American Thyroid Association (ATA) criteria, and cases with no preconception TSH levels were excluded from the analysis. We calculated medians of TSH levels in each month for each trimester and also similarly grouped them. To compare baseline characteristics as well as maternal outcomes between preconception and trimester-wise TSH groups, re-coding of data was performed. Pregnancy outcomes were grouped as pregnancy loss (including abortion, ectopic pregnancy, termination of pregnancy and intrauterine death or stillbirth) and uneventful pregnancy (live births, twin and triplet pregnancies). Similarly, the etiology of hypothyroidism was divided into autoimmune and post-treatment categories. Univariate analysis of baseline characteristics was performed for preconception TSH groups. Subgroup analysis of maternal outcomes of pregnancies affected by uncontrolled hypothyroidism during preconception and trimester-wise gestational periods was assessed using the Chi-square and Fischer Exact test. Statistical significance was taken as *P*-value ≤0.05. Data analysis was performed on Statistical Package for the Social Sciences version 20.0.

## Results

**Fig. 1:**
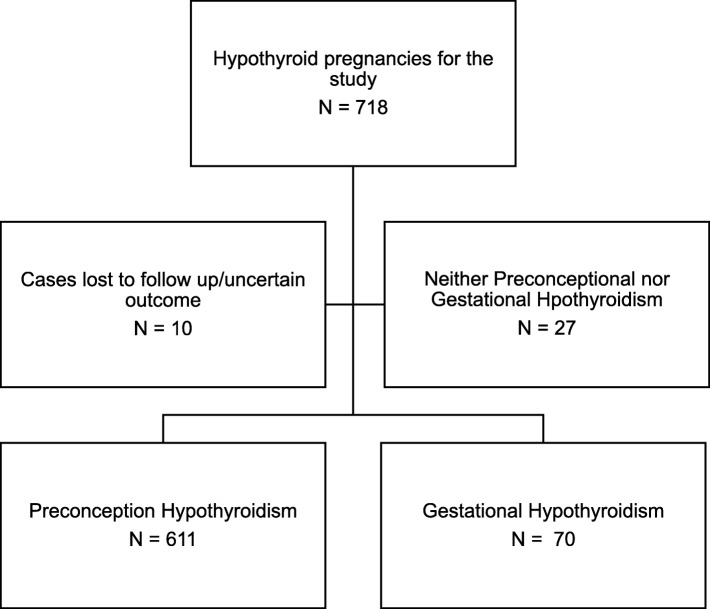
Flow chart of the study. Number of patients in preconception and gestational hypothyroid groups

During 2008–2016, a total of 718 cases were retrieved using the code hypothyroidism and pregnancy out of which 10 cases were either lost to follow up or delivered elsewhere. Figure 1 shows groups of hypothyroid patients analyzed. Six patients had delivered twice during this period, so 702 hypothyroid patients out of 708 pregnancies were included in the study population.

### Characteristics of hypothyroid pregnancies and maternal outcomes

Table 1 summarizes the clinical characteristics of overall hypothyroid pregnancies. The mean age of the hypothyroid pregnancies was 31 years (SD 4.73). These women had presented at various gestational ages in our hospital, the majority presenting in the first trimester (61%) with a maximum of 9.6% present in the 8th week. Most of the patients were diagnosed before pregnancy with unknown initial severity of hypothyroidism, however, there were more subclinical cases compared to overt hypothyroidism even in those diagnosed during pregnancy. Twenty-seven cases did not classify into either pre conceptional or gestational hypothyroidism. Three cases with Hashimoto’s also had a goiter. Family history of hypothyroidism was positive in 15.3% (10.9% in first degree relatives and 3.5% in second-degree relatives), whereas it was negative in 43% cases. TSH levels were determined in 53.2, 56.7, 61.7 and 66.6% of cases in preconception, 1st, 2nd, and 3rd trimester periods respectively. Median TSH (with interquartile range) before conception was 2.9 (1.5–5.8), whereas, during first, second and third trimester, it was 3.0 (1.4–5.4), 2.4 (1.5–3.8) and 2.3 (1.5–3.8) respectively.

**Table 1:** Clinical features of overall hypothyroid pregnancies at Aga Khan University

Clinical characteristics	Frequency (%) *N* = 708
Age (years)	
Age 18–25	87 (12.3)
Age 26–33	403 (56.9)
Age 34–40	200 (28.2)
Age 41–47	18 (2.5)
Parity	
Nulliparous	216 (31.4)
Primiparous	221 (32.2)
Biparous	140 (20.4)
Multiparous	110 (16.0)
Missing	21 (3.0)
Body Mass Index (Kg/m^2^)	
Underweight (< 18.5)	19 (2.7)
Normal (18.5–24.9)	177 (25.0)
Overweight (25–29.9)	221 (31.2)
Obesity class I (30–34.9)	150 (21.2)
Obesity class II and above (> = 35)	47 (6.6)
Unknown	94 (13.3)
Gestational age at antenatal visit (weeks of pregnancy)	11 (7–19)
Mode of labor (for live births only; *N* = 638)	
Spontaneous	145 (22.7)
Induced	157 (24.6)
Augmented	76 (11.9)
Not applicable (because of caesarian section)	260 (40.8)
Mode of delivery (for live births only; N = 638)	
Em-LSCS	149 (23.4)
LSCS	223 (35.0)
Instrumental	23 (3.6)
SVD	87 (13.6)
SVD with Episiotomy	156 (24.5)
Gestational age at delivery (weeks)	38 (37–39)
Hypothyroidism diagnosed during pregnancy	
Uncategorized	14 (2.0)
Overt hypothyroidism	13 (1.8)
Subclinical hypothyroidism	43 (6.1)
Hypothyroidism diagnosed prior to pregnancy	
Uncategorized	308 (43.5)
Overt hypothyroidism	86 (12.6)
Subclinical hypothyroidism	217 (30.6)
Neither preconceptional nor Gestational Hypothyroidism	27 (3.8)
Etiology of Hypothyroidism	
Hashimoto’s	183 (25.8)
Post Radioactive Iodine ablation	8 (1.1)
Post-surgical	6 (0.8)
Postpartum thyroiditis	4 (0.6)
Secondary Hypothyroidism	1 (0.1)
After medical treatment for Grave’s Disease	2 (0.3)
Congenital	1 (0.1)
Nodular Goiter	2 (0.3)
Subacute thyroiditis	1 (0.1)
Unknown	500 (70.6)
Thyroid antibodies	
Positive	158 (22.3)
Negative	57 (8.1)
Not checked	493 (69.6)
Comorbidities (positive cases only)	
Diabetes mellitus	55 (7.8)
Gestational diabetes	150 (21.2)
Hypertension	
*Pregnancy Induced Hypertension*	75 (10.6)
*Chronic Hypertension*	34 (4.8)
Smoking	1 (0.1)

Data are expressed as mean (± standard deviation) or median (interquartile range)

A total of 372 pregnancies delivered through cesarean section (C-section). Out of those who underwent elective lower segment cesarean section (LSCS), 60.5% did so because of previous C-section and the rest have appropriate obstetric indications (6.3% fetal growth restriction or intrauterine growth restriction [IUGR], 4.0% twin pregnancy, 3.6% failure to progress amongst others) whereas, 4.0% were due to patient’s request. Twenty-three percent of pregnancies underwent emergency LSCS (Em-LSCS), out of which 22.3% were due to non-reassuring cardiotocography (CTG), 4.7% due to pre-eclampsia, and 4.1% due to IUGR amongst others.

In our study population, postpartum hemorrhage (PPH) was the most frequent maternal outcome (38.8%) where 61.5% of cases resulted in a PPH blood volume of 500 ml. We found gestational hypertension (11.6%) and pre-eclampsia (4.1%) to be the next most common maternal outcomes. The overall pregnancy loss occurred in 70 cases with abortions in 6.5% of pregnancies.

### Association between preconception and gestational (trimester-wise) TSH and baseline characteristics as well as maternal outcomes

Details of clinical characteristics and maternal outcomes are described for these patients in Table 2.

**Table 2:** Clinical features and maternal outcomes of hypothyroid pregnancies who had TSH measured before conception and during each trimester at Aga Khan University

	Preconception TSH data	1st Trimester TSH data	2nd Trimester TSH data	3rd Trimester TSH data
CLINICAL CHARACTERISTICS	N (%) *N* = 377	N (%) *N* = 402	N (%) *N* = 437	N (%) *N* = 471
Age (years)				
18–25	31 (8.22)	47 (11.7)	57 (13.0)	60 (12.7)
26–33	221 (58.62)	234 (58.2)	253 (57.9)	271 (57.5)
34–40	119 (31.56)	111 (27.6)	121 (27.7)	131 (27.8)
41–47	6 (1.59)	10 (2.5)	6 (1.4)	9 (1.9)
Parity				
Nulliparous	96 (25.81)	126 (31.3)	146 (33.4)	147 (31.2)
Multiparous	276 (74.19)	269 (66.9)	285 (65.2)	312 (66.2)
Body Mass Index (Kg/m^2^)				
Underweight (< 18.5)	13 (3.83)	13 (3.2)	16 (3.7)	14 (3.0)
Normal (18.5–24.9)	99 (29.20)	118 (29.4)	124 (28.4)	120 (25.5)
Overweight (25–29.9)	116 (34.22)	124 (30.8)	141 (32.2)	138 (29.3)
Obesity class I (30–34.9)	88 (25.96)	81 (20.1)	95 (21.7)	103 (21.9)
Obesity class II and above (> = 35)	23 (6.78)	28 (7.0)	28 (6.4)	38 (8.1)
Hypothyroidism diagnosed during pregnancy				
Yes	15 (4.00)	38 (9.5)	45 (10.3)	46 (9.8)
No	360 (96.00)	361 (89.8)	387 (88.6)	413 (87.7)
Etiology of Hypothyroidism				
Autoimmune	362 (96.28)	390 (97.0)	425 (97.3)	457 (97.0)
Post treatment^a^	14 (3.72)	12 (3.0)	12 (2.7)	14 (3.0)
Thyroid antibodies				
Positive	114 (78.08)	114 (28.4)	115 (26.3)	116 (24.6)
Negative	32 (21.92)	30 (7.5)	42 (9.6)	43 (9.1)
Comorbidities				
Diabetes Mellitus				
yes	28 (7.43)	34 (8.5)	31 (7.1)	34 (7.2)
no	349 (92.57)	368 (91.5)	406 (92.9)	437 (92.8)
Gestational diabetes mellitus				
yes	82 (21.75)	82 (20.4)	99 (22.7)	123 (26.1)
no	295 (78.25)	320 (79.6)	338 (77.3)	348 (73.9)
Hypertension				
Normotensive	327 (86.74)	353 (87.8)	366 (83.8)	396 (84.1)
Gestational Hypertension & Chronic Hypertension	50 (13.26)	49 (12.2)	71 (16.2)	75 (15.9)
MATERNAL OUTCOMES				
Mode of labor (for live births only)				
Spontaneous	66 (33.00)	75 (18.7)	86 (19.7)	94 (20.0)
Induced	89 (44.50)	84 (20.9)	110 (25.2)	123 (26.1)
Augmented	45 (22.50)	44 (10.9)	55 (12.6)	58 (12.3)
Not applicable due to Caesarean section	145 (38.5)	151 (37.6)	171 (39.1)	195 (41.4)
Mode of delivery (for live births only)				
Em-LSCS	73 (19.4)	72 (17.9)	92 (21.1)	109 (23.1)
LSCS	131 (34.7)	134 (33.3)	148 (33.9)	168 (35.7)
Instrumental	13 (3.77)	14 (3.5)	18 (4.1)	22 (4.7)
SVD	128 (37.10)	134 (33.3)	164 (37.5)	171 (36.3)
Pregnancy outcomes				
Abortion	27 (7.16)	33 (8.2)	4 (0.9)	N/A
Live Birth (Twins/Triplets)	344 (91.25)	355 (88.3)	422 (96.5)	471 (100)
Ectopic	1 (0.27)	4 (1.0)	0 (0)	N/A
Termination of Pregnancy	1 (0.27)	5 (1.2)	5 (1.1)	0 (0)
Intrauterine Death/Stillbirth	4 (0.01)	5 (1.2)	6 (1.4)	0 (0)
Gestational Hypertension				
Yes	34 (9.0)	N/A	52 (11.9)	56 (11.9)
No	343 (91.0)		385 (88.1)	415 (88.1)
Pre-Eclampsia				
Yes	10 (2.7)	N/A	19 (4.3)	20 (4.2)
No	367 (97.3)		418 (95.7)	451 (95.8)
Postpartum Hemorrhage				
Yes	151 (40.1)	N/A	178 (40.7)	204 (43.3)
No	226 (59.9)		259 (59.3)	267 (56.7)
Placental abruption				
No	377 (100)	N/A	437 (100)	1 (0.2)
Yes	0 (0)		0 (0)	470 (99.8)

*TSH* Thyroid stimulating Hormone. *N/A* not applicable. *LSCS* Lower segment Caesarean section. *Em-LSCS* emergency Lower segment Caesarean section. *SVD* Spontaneous vaginal delivery

^a^Post medical treatment for Grave’s disease, Post-Radioactive Iodine Ablation and Post-Surgical

We did not find any significant association between baseline characteristics and preconception TSH groups (Table 3). Subgroup analysis of maternal outcomes revealed a significant association of mode of delivery including cesarean section (emergency & elective) and postpartum hemorrhage with a TSH value of more than 2.5 uIU/mL before pregnancy (*p* values ≤0.05). Similarly, postpartum hemorrhage is also significantly associated with a TSH value of more than 2.5 uIU/mL in the third trimester (*p*-value 0.03) (Table 4). However, there is no significant effect of TSH levels during each trimester on other maternal outcomes, and the median TSH remained below 2.5 uIU/mL except in the first trimester only [31, 38].

**Table 3:** Comparison of baseline characteristics and maternal outcomes of controlled and uncontrolled hypothyroid patients before conception

CLINICAL CHARACTERISTICS	TSH PRECONCEPTION		*P* -value
	Controlled (up to 2.5 mIU/L) *N* = 170	Uncontrolled (greater than 2.5 mIU/L) *N* = 207	
	N (%)	N (%)	
Age (years)			
18–25	15 (3.98)	16 (4.24)	0.43^a^
26–33	104 (27.59)	117 (31.03)	
34–40	50 (13.26)	69 (18.30)	
41–47	1 (0.27)	5 (1.33)	
Parity			
Nulliparous	42 (11.29)	54 (14.52)	0.8
Multiparous	125 (33.60)	151 (40.59)	
Body Mass Index (Kg/m2)			
Underweight (< 18.5)	7 (2.06)	6 (1.77)	0.2
Normal (18.5–24.9)	45 (13.27)	54 (15.93)	
Overweight (25–29.9)	56 (16.52)	60 (17.70)	
Obesity class I (30–34.9)	31 (9.14)	57 (16.81)	
Obesity class II and above (> = 35)	13 (3.83)	10 (2.95)	
Etiology of Hypothyroidism			
Autoimmune	163 (43.35)	199 (52.93)	0.7
Post treatment^$^	7 (1.86)	7 (1.86)	
Thyroid antibodies			
Positive	54 (36.99)	60 (41.10)	0.7
Negative	14 (9.59)	18 (12.33)	
*COMORBIDITIES*			
Diabetes Mellitus			
Yes	10 (2.65)	18 (4.77)	0.3
No	160 (42.44)	189 (50.13)	
Gestational diabetes mellitus			
Yes	38 (10.08)	44 (11.67)	
No	132 (35.01)	163 (43.24)	0.8
Hypertension			
Normotensive	151 (40.05)	176 (46.68)	
Previous Gestational Hypertension/Chronic Hypertension	19 (5.04)	31 (8.22)	0.3
MATERNAL OUTCOMES			
Mode of labor (for live births only)			
Spontaneous	29 (14.50)	37 (18.50)	0.24
Induced	45 (22.50)		
Augmented	27 (13.50)	44 (22.00)	
Not applicable due to Caesarean section		18 (9.00)	
Mode of delivery (for live births only)			
LSCS	80 (23.19)	124 (35.94)	0.012
Instrumental	7 (2.03)	6 (1.74)	
SVD	71 (20.58)	57 (16.52)	
Pregnancy outcomes			
Abortion	12 (3.18)	15 (3.98)	0.9^a^
Live Birth (Twins/Triplets)	157 (41.64)	187 (49.60)	
Ectopic	0 (0.00)	1 (0.27)	
Termination of Pregnancy	0 (0.00)	1 (0.27)	
Intrauterine Death/Stillbirth	1 (0.27)	3 (0.80)	
Gestational Hypertension			
Yes	13 (7.6)	21 (10.1)	0.4
No	157 (92.4)	186 (89.9)	
Pre-Eclampsia			
Yes	2 (1.2)	8 (3.9)	0.19^a^
No	168 (98.8)	199 (96.1)	
Postpartum Hemorrhage			
Yes	56 (32.9)	95 (45.9)	0.01
No	114 (67.1)	112 (54.1)	

^a^Fischer’s Exact

^$^Post medical treatment for Grave’s disease, Post-Radioactive Iodine Ablation and Post-Surgical

*LSCS* Lower segment Caesarean section. *SVD* Spontaneous vaginal delivery

**Table 4:** Univariate analysis of maternal outcomes between TSH groups in 1st, 2nd and 3rd trimesters

	1st Trimester TSH data			2nd Trimester TSH data			3rd Trimester TSH data		
	Controlled (up to 2.5 mIU/L) *N* = 170	Uncontrolled (greater than 2.5 mIU/L) *N* = 207	*P* -value	Controlled (up to 2.5 mIU/L) *N* = 230	Uncontrolled (greater than 2.5 mIU/L) *N* = 207	*P* -value	Controlled (up to 2.5 mIU/L) *N* = 256	Uncontrolled (greater than 2.5 mIU/L) *N* = 215	*P* -value
Pregnancy Loss	12 (6.9%)	26 (11.4%)	0.12	7 (3.0)	7 (3.4)	0.8	0 (0)	0 (0)	**–**
Uneventful pregnancy (Live births & twins/triplets & TOP)	162 (93.1%)	202 (88.6%)		223 (97.0)	200 (96.6)		256 (100.0)	215 (100.0)	
Gestational Hypertension	N/A	N/A				0.13			
Yes				22 (9.6)	30 (14.5)		25 (9.8)	31 (14.4)	0.12
No				208 (90.4)	177 (85.5)		231 (90.2)	184 (85.6)	
Pre-Eclampsia	N/A	N/A				0.35			0.08
Yes				12 (5.2)	7 (3.4)		7 (2.7)	13 (6.0)	
No				218 (94.8)	200 (96.6)		249 (97.3)	202 (94.0)	
Postpartum Hemorrhage	N/A	N/A				0.09			0.03
Yes				85 (37)	93 (44.9)		99 (38.7)	105 (48.8)	
No				145 (63)	114 (55.1)		157 (61.3)	110 (51.2)	
Placental abruption	N/A	N/A				–			0.5 ^a^
No				230 (100)	207 (100)		256 (100)	214 (99.5)	
Yes				0 (0)	0 (0)		0 (0)	1 (0.5)	

*TOP* Termination of pregnancy

^a^Fischer’s Exact

## Discussion

Maternity care is different in rural and urban communities of Pakistan, where health facility-related births are limited mostly to urban setup [39]. However access to such healthcare has overall increased to almost 3.6 times from 1991 to 2013, but the government goals are still not achieved [39]. Rural health centers, dispensaries, basic health units, and the lady health worker program in Pakistan has allowed more home-based deliveries as compared to health facility births in less privileged areas [40]. This can be one reason why all hypothyroid pregnant women do not consider antenatal care or rather never access a tertiary hospital. Although, majority of pregnant women in our study were diagnosed with hypothyroidism before conception (89.7%), even then the number of TSH assays performed was less in our cohort (53.2, 56.7, 61.7 and 66.6% of cases in preconception, 1st, 2nd, and 3rd trimester periods), in contrast to a Scottish study [41]. Although the etiology of hypothyroidism remained unknown in the majority of cases, we found that the most common cause of hypothyroidism was autoimmune thyroiditis in our population which is in accordance with a similar study reported in the literature [42]. Other causes included radioiodine ablation, post-surgical hypothyroidism, and postpartum thyroiditis. Our study found 22.3% of the women to be anti-TPO positive amongst hypothyroid pregnancies. The presence of thyroid anti-TPO antibodies were found positive in 40% of hypothyroid pregnant females in one of the epidemiological study [7], while another study reported 57.1% in subclinical hypothyroid cases [43]. Whether all women should be checked for autoimmunity once diagnosed with hypothyroidism remains an understudied area.

Data regarding maternal comorbidities affecting hypothyroidism is scarce and controversial in the literature. A study on Bangladeshi pregnant women concluded that cases with overt hypothyroidism were prone to have gestational hypertension (GH) (42.9%) and gestational diabetes (38.1%) as compared to subclinical cases. A study on more than 5000 pregnancies from Finland reported that overt hypothyroidism predicts the risk of developing diabetes later [hazard ratio (HR) 6.0 (95% confidence interval) (2.2–16.4)] [44]. In our population, gestational diabetes was present in 21.2% cases and GH was only 10.6%. We need more prospective longitudinal studies to analyze this difference from our center.

In our study Subclinical hypothyroidism was present in around 37% of our total hypothyroid pregnant cases. A retrospective cohort study based on 500 pregnant women in the Indian city of Chennai conducted in 2007, reported 2.8% subclinical hypothyroidism [43], and a prospective study from Iran reported 11.3% of subclinical hypothyroidism whereas clinical hypothyroidism was present in 2.4% pregnant females amongst 600 women with singleton pregnancies [45]. A large study from the United Kingdom (UK) database reported 7.4% subclinical hypothyroidism in women already on levothyroxine replacement, with increased risk of miscarriages as TSH rises above 2.5 uIU/mL [46]. We also observed 14% overt hypothyroidism in our study. A cross-sectional multicentre study of different states of India reported an overall 36.07% prevalence of hypothyroidism in pregnancy according to ATA cut-offs [7]. Although we could not classify a substantial number of patients as overt or subclinical in our population of 708 hypothyroid pregnancies, amongst those clearly labeled, it consisted mainly of subclinical hypothyroidism.

Most of the findings on maternal outcomes from our study are consistent with that conducted in other parts of the world [13, 45]. Our study demonstrates that TSH > 2.5 uIU/mL does not have a significant relationship with abortions or any cause of pregnancy loss (*p*-value 0.9 for preconception TSH and *p* values 0.12 & 0.8 for 1st and 2nd trimester TSH respectively), similar to some recently published studies [47, 48]. On the other hand, there is a significant relationship between preconception TSH > 2.5 uIU/mL with cesarean section in our cohort (*p*-value 0.047), which is different from a small retrospective analysis of 167 pregnancies in the UK [1]. Although the overall C-section rate remained high in their study cohort compared to the hospital as well as the total UK C-section rate. This is in contrast to a study from China where preconception TSH > 2.5 uIU/mL is associated with adverse pregnancy outcomes including cesarean section rate (aOR: 1·15, 95% CI: 1·10–1·22) [49]. GH (*p*-value 0.4) and pre-eclampsia (p-value 0.19) were not significantly associated in our patients with preconception TSH > 2.5 uIU/mL. This is similar to a case-control study conducted in India, where gestational hypertension and pre-eclampsia were not associated with hypothyroidism, although their TSH cutoff was between 3.1–6.2 uIU/mL [50] and a population-based prospective data from Finland also reports no significant association [subclinical hypothyroidism 20/213 (9.4%), p-value 0.615, HR 1.1 (0.7–1.7), Adjusted HR 1.0 (0.7–1.6)] [44]. In our study, postpartum hemorrhage was significantly associated with hypothyroidism (p-value 0.01 in preconception and 0.03 in 3rd trimester), however, this is less well reported in the literature [32, 51] and need multiple prospective case-control studies in our setup. In a Turkish study, which was conducted on a well-defined number of patients, taking into account several factors for the cause of bleeding, no difference was found in terms of coagulation parameters and PPH between euthyroid, subclinical hypothyroid and healthy controls [52].

Internationally defined controlled levels of TSH (≤ 2.5 uIU/mL) during each trimester did not show significant association with the outcomes in our study except for mode of delivery (cesarean section) in preconception and postpartum hemorrhage in the preconception and third trimester. We need to analyze whether the absence of availability of trimester-specific TSH ranges in our region is the reason behind this result and this demands a large scale population based study of all pregnant women in our country.

There are several limitations to our study, the most important being that it was a retrospective analysis with TSH levels not measured according to general recommendations, despite an adequate diagnosis and levothyroxine replacement timely initiated. Further, the results of this study cannot be generalized to all pregnant populations as screening for thyroid dysfunction is not universally performed and not all females with hypothyroidism would have attended the antenatal clinic despite an early miscarriage. Similarly, the autoimmune status of the pregnant population need to be assessed through population-based studies as anti-TPO antibodies are not routinely measured in our setting. This study, however, can be regarded as a baseline reflection of our hypothyroid pregnant population. Moreover, this is the only study with a large number of patients (708) from Pakistan. We therefore recommend, large prospective and multicentre, studies to assess the strength of associations between maternal hypothyroidism and outcome variables which may contribute towards unifying universal screening and follow up management of hypothyroid pregnancies.

## Conclusion

The important findings of this study were excessive post-partum hemorrhage and elective as well as emergency C-sections. Postpartum hemorrhage had a significant association with uncontrolled TSH before conception and in the third trimester. Moreover, this study observed more subclinical hypothyroidism in the peri-gestational period, which poses a great potential of adversely affecting maternal outcomes.

## Supplementary information

**Additional file 1: Figure S1.** Schematic division of controlled and uncontrolled TSH groups in preconception and gestational periods. TSH units in mIU/L

## Data Availability

The dataset supporting the findings of this study can be made available upon request to the first author whose email is drzareenkiran@gmail.com.

## Abbreviations

C-section: Caesarean section
CTG: Cardiotocography
FT4: Free Thyroxine
IUD: Intrauterine death
IUGR: Intrauterine growth retardation
LSCS: Lower segment cesarean section
PPH: Postpartum hemorrhage
TPO: Thyroid peroxidase
TSH: Thyroid Stimulating Hormone
