# Supplementary material for: Maternal characteristics and outcomes affected by hypothyroidism during pregnancy (maternal hypothyroidism on pregnancy outcomes, MHPO-1)
Source: BMC Pregnancy Childbirth. 2019 Dec 5;19:476. doi: 10.1186/s12884-019-2596-9 (PMC6896307; doi:10.1186/s12884-019-2596-9)
Supplement: Supplementary file 1 — Additional file 1: Figure S1. Schematic division of controlled and uncontrolled TSH groups in preconception and gestational periods. TSH units in mIU/L [file 12884_2019_2596_MOESM1_ESM.docx]

**Figure S1. Schematic division of controlled and uncontrolled TSH groups in preconception and gestational periods. TSH units in mIU/L.**
